# Choline derivatives immobilized on silica to catalyze transesterification reaction for production of glycerophosphocholine

**DOI:** 10.3906/kim-1907-43

**Published:** 2020-02-11

**Authors:** Longhui WEI, Binglin LI, Xiaoli ZHANG, Kai TANG, Wenwen ZHENG, Dan CHEN, Binxia ZHAO

**Affiliations:** 1 Department of Chemical Engineering, Northwest University, Xi’an, Shaanxi P.R. China; 2 College of Food Science and Technology, Northwest University, Xi’an, Shaanxi P.R. China

**Keywords:** Choline derivatives, glycerophosphocholine, transesterification reaction, immobilized choline derivative, kinetic

## Abstract

Choline derivatives were covalently immobilized on the surface of γ -aminated silica. The obtained immobilized choline derivative was then successfully used for a transesterification reaction to produce glycerophosphocholine (GPC). Fourier transform infrared analysis and thermogravimetric analysis/differential thermal gravimetry indicated that the surface of the γ -aminated silica was covered by choline derivatives and the highest immobilization amount reached 1.1 mmol/g under optimal conditions. More importantly, the highest yield of GPC reached 97.9% during transesterification. With regard to GPC in food or medicine for human use, the immobilization technology can avoid catalyst contamination of the product and increase the safety of the product. The recyclability and stability of the immobilized choline derivative were excellent, as demonstrated by its use 20 times without any loss of productivity. A first-order kinetic model was employed and the relevant parameters were calculated to investigate kinetic characteristics of transesterification.

## 1. Introduction

L-α-Glycerophosphocholine (GPC) has many applications in the pharmaceutical and food industries [1,2]. According to the US Food and Drug Administration, GPC is listed as one of safe snack foods [3]. Clinical studies have reported that GPC has a positive therapeutic effect on brain diseases such as Alzheimer disease and Epilepsy [4–6]. Moreover, GPC can directly elevate acetylcholine levels in the hippocampus and increase the activity of the cholinergic neurotransmission to improve cognitive performances in stroke patients [7,8].

Previously, GPC was extracted from plant or animal tissues [9,10], which required the use of many toxic organic solvents. However, GPC is a kind of naturally rare phospholipid. The low availability and complicated technology result in its very high price. So far, commercial GPC is mainly prepared by artificial synthesis. Phospholipase A1 was employed as the biocatalyst for the hydrolysis of phosphatidylcholine (PC) to produce GPC [3]. However, the fragile nature and high cost of the biocatalyst limit its industrial applications. It was reported that GPC was synthesized through a transesterification reaction by metal catalyst (chromic chloride) [11–13]. Residues of toxic metal ions and the unsatisfactory yield were limiting factors in industrial applications. Recently, the widely used catalysts for the synthesis of GPC are organic alkalis including choline [14], tetrabutylammonium hydroxide [15–17], and sodium methoxide [18,19]. Among those, choline showed the highest activity and selectivity. However, after the reaction, choline was very hard to separate with GPC from the mixture in the actual production process, due to similar physical and chemical properties.

Therefore, many investigations have paid attention to the development of the ideal reaction system for transesterification, which should be an efficient, cheap, nontoxic, biocompatible, and facile separation system. For example, GPC was synthesized by the quaternary ammonium base resin [2]. After the reaction, the catalyst could be easily separated and recycled. However, the catalyst could not tolerate the higher temperature ofreaction. Additionally, the resulting GPC might be contaminated by the toxic catalyst. Next, calcined sodium silicate could serve as a heterogeneous catalyst for the production of GPC [20], but complex preparation processes limit the use of the catalyst.

In this study, choline derivatives were covalently immobilized to the surface of γ -aminated silica. The obtained immobilized choline derivative was successfully used for the hydrolysis of PC to produce GPC. After immobilization, the catalyst (choline derivative) could be easily collected and reused to avoid the contamination of the product, improve the safety of the product, and reduce the production cost. A first-order kinetic model was employed and the relevant parameters were calculated to investigate the kinetic characteristics of transesterification. The excellent results show that this technology is a more promising candidate for the industrial production of GPC.

## 2. Experimental

### 2.1. Materials

Glycerol phosphatidylcholine (GPC) and phosphatidylcholine (PC) were purchased from Sigma-Aldrich Co. (St. Louis, MO, USA). Silica 60H was obtained from Qingdao Haiyang Chemical Co. Ltd.(China). γ - Aminopropyltriethoxysilane (KH550) and other reagents were purchased from Sinopharm Chemical Reagent Co., Ltd. All other reagents were of standard laboratory grades.

### 2.2. Catalyst preparation

Five grams of trimethylamine salt was reacted with 10 mL of ethanol at 30 °C. Then 3.5 mL of epichlorohydrin was added dropwise in 1 h. The pH value of the reaction solution was adjusted to 8.0 via 1 M NaOH solution and it was incubated at 40 °C for 3 h at 500 rpm. After washing three times with acetone and twice with deionized water, the crystals (choline derivative) were dried at 50 °C under vacuum conditions.

Silica (2 g) was added to 20 mL of toluene, and then 1.5 mL of γ -aminopropyltriethoxysilane (KH550) was added to react at 80 °C for 8 h at 500 rpm. The resulting γ -aminopropylated silica (SG-NH2) was collected by centrifugation, which was washed with anhydrous ethanol three times and deionized water twice. Then the nanoparticles were dried at 50 °C under vacuum conditions.

One gram of SG-NH2 was added to 20 mL of deionized water, and then 0.2 g of choline derivative was added to react for 7 h at 30 °C in nitrogen atmosphere at 500 rpm. NaOH (1 M, 3 mL) was added to the reaction solution and the reaction was stirred at room temperature for 1 h. The resulting precipitates were washed at least three times with deionized water and subjected to centrifugation. The resulting precipitates (immobilized choline derivative) were dried at 50 °C under vacuum conditions and the basicity of the catalyst was determined [21,22].

### 2.3. Catalyst characterizations

The amount of immobilized choline derivative was measured by thermogravimetric analysis (TGA) using a thermogravimetric analyzer (STA449F3, Netzsch, Germany) under nitrogen flow at a heating rate of 10 K/min from room temperature to 800 °C. Fourier transform infrared (FT-IR) spectroscopy was performed on a Fourier transform infrared spectrometer (Frontier, PerkinElmer, USA).

### 2.4. Interesterification procedure

For interesterification, PC (0.0790 g) was added to 10 mL of anhydrous ethanol, and then 0.2 g of the immobilized choline derivative was added to react for 4 h at 45 °C at 250 rpm. Samples (30 μL) were taken at 30-min intervals for 4 h, and a standard sample of GPC was prepared by interesterification and purified by our lab to be used in the analysis of high-performance liquid chromatography (HPLC) [3].

### 2.5. Determination of kinetic parameters for free and immobilized choline derivative

The assumption employed in this work with the first-order reaction kinetics was that the effect of ethanol could be neglected under the optimum concentration of PC, which was determined by a preliminary experiment. To determine the respective kinetic parameters of free and immobilized choline derivative at the optimum operational conditions, initial reaction rates were measured for concentrations of PC under different temperatures, according to the method described above. The parameters K and Ea were estimated by performing a nonlinear fit of the experimental values to the first-order kinetic model of Eq. (1):

(1)-ln(1-x)=kt

where x is the catalytic reaction conversion rate and t (min) is the reaction time.

The calculation of the activation energy of the reaction was calculated via the Arrhenius equation:

(2)lnk=lnA-Ea/RT

where k (min ^-1^ ) is the catalytic reaction rate constant, Ea (kJ/mol) is the reaction activation energy, A is the preexponential factor, T (Kelvin) is the reaction temperature, and R (J/mol K) is the molar gas constant.

All data are the average values of triple experiments, and error bars represent standard error of the mean.

## 3. Results and discussion

### 3.1. Catalyst preparation

**Figure 1:**
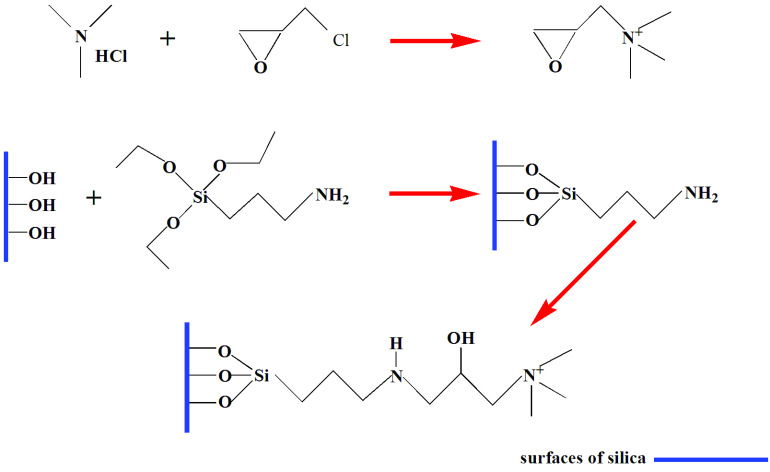
Preparation of immobilized choline derivative.

The immobilization of the choline derivative was a three-step process (Figure 1). First, choline derivatives were synthesized by trimethylamine hydrochloride and epichlorohydrin. Second, KH550 was covalently coupled on the surface of silica for the preparation of γ -amino-functionalized particles (γ -aminated silica). Third, choline derivatives were used to react with the coupled amino group and covalently bonded to the surface of silica to obtain the immobilized choline derivative.

**Figure 2:**
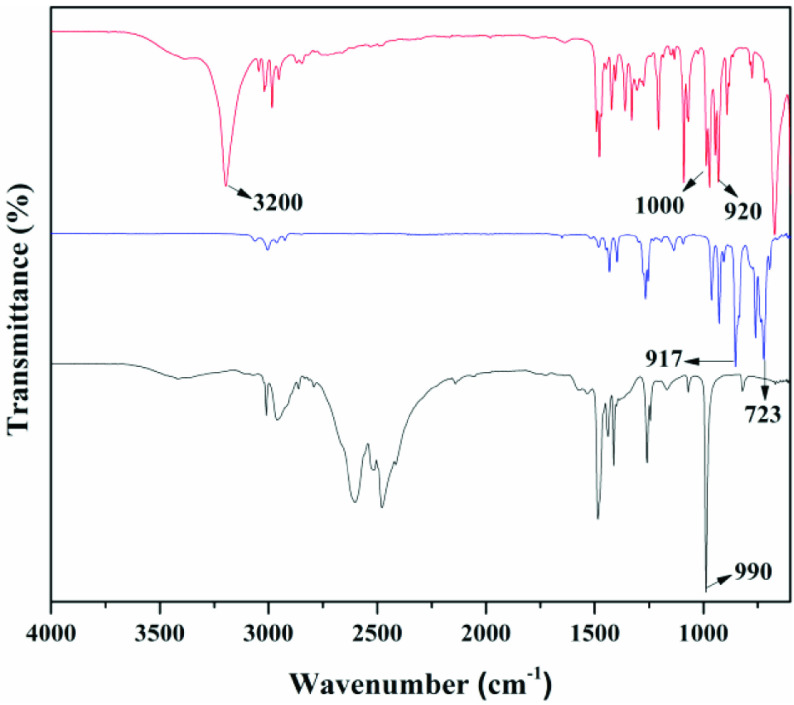
FTIR-ATR spectra of choline derivative - red line, trimethylamine hydrochloride - black line, and epichlorohydrin - blue line.

Figure 2 shows the FT-IR spectra of epichlorohydrin (red line), trimethylamine hydrochloride (black line), and choline derivative (blue line). Two main bands were observed in the epichlorohydrin: the peak at about 723 cm ^-1^ , attributed to the C-Cl stretching vibration, and a band peaking at 917 cm ^-1^ corresponding to the epoxy group [23]. The main peak of trimethylamine hydrochloride at about 990 cm ^-1^ was due to the C-N of -N(CH _3_ ) _3_ [24]. After reaction, the band at 910–920 cm ^-1^ attributed to the epoxy group and the broad band at 990–1020 cm ^-1^ ascribed to C-N of -N(CH _3_ ) _3_ of choline derivative could be observed. The broad band at 3200 cm ^-1^ might be attributed to the O-H stretching vibration of the crystallized water molecules that remain after drying. Similar phenomena could be found in many previous studies [25,26]. The results indicated that the choline derivative was successfully prepared. The purity of our prepared choline derivative was detected by several methods. First, acid-base titration was employed to measure and calculate the molar mass [27,28]. The choline derivative’s relative molecular mass was 151.51 g/mol, which was very close to the theoretical value (151.63 g/mol). Then the melting point of the prepared choline derivative was measured by micro melting point apparatus. The detection results showed that the melting point of the substance was 138.7 °C. The reported standard choline derivative’s melting point was 140 °C [29,30].

**Figure 3:**
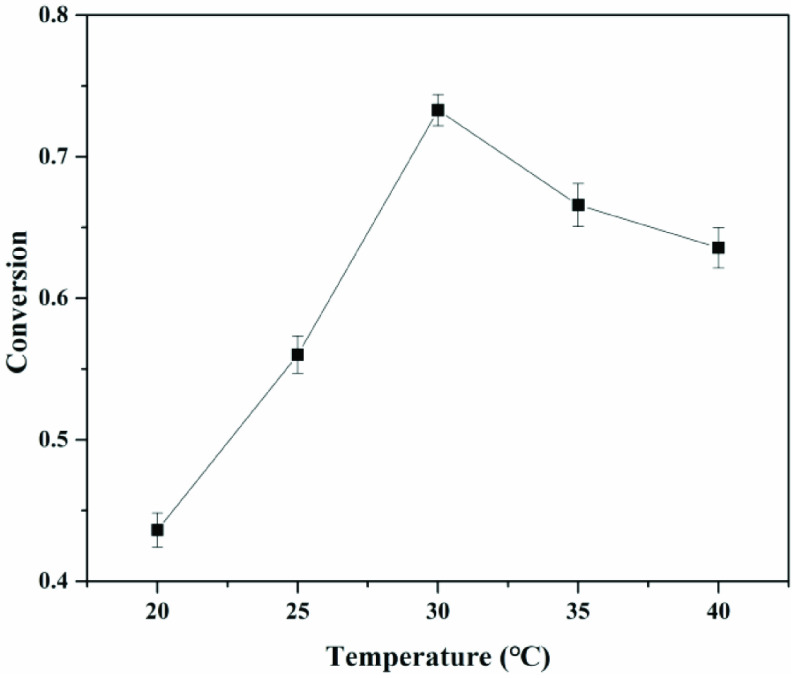
The optimal reaction temperature for preparing catalyst.

Next, variables affecting the immobilized choline derivative were systematically investigated. Previous studies indicated that the reaction temperature was the crucial factor for immobilization [27,29]. As shown in Figure 3, it was observed that for reaction temperatures below 30 °C, the immobilization yield increased with the operational temperature. For temperatures above this value, a decrease in the immobilized yield was observed. With increasing operational temperature, more collisions between the choline derivative and carriers were expected to occur, and the energy of the choline derivative also increased, which was beneficial for forming more immobilized choline derivative. When the temperature was very high, the epoxy group of choline derivatives would be broken, reducing the activity of choline derivatives and the relevant immobilization yield.

### 3.2. Catalyst characterization

**Figure 4:**
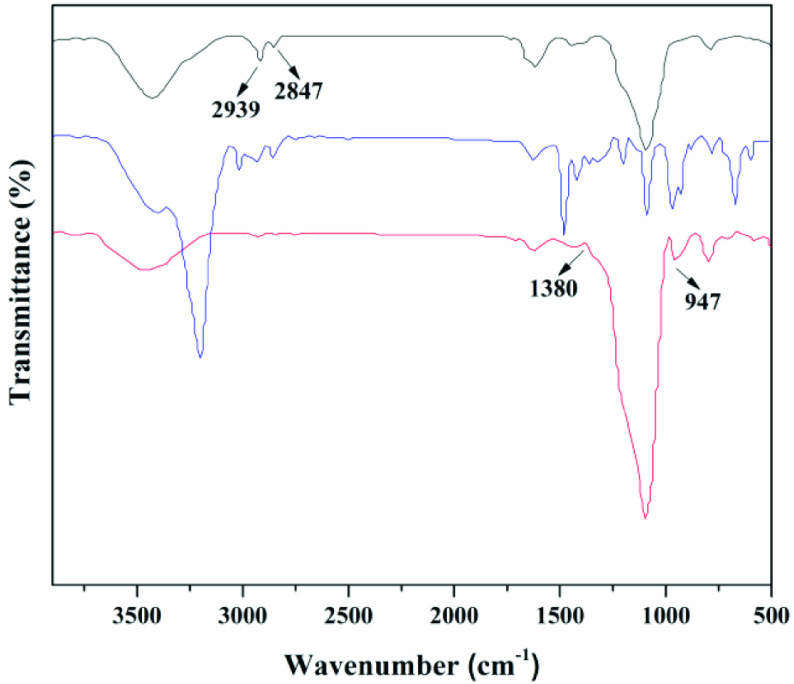
FTIR-ATR spectra of choline derivative - blue line, SG-NH2 - black line, and immobilized choline derivative - red line

**Figure 5:**
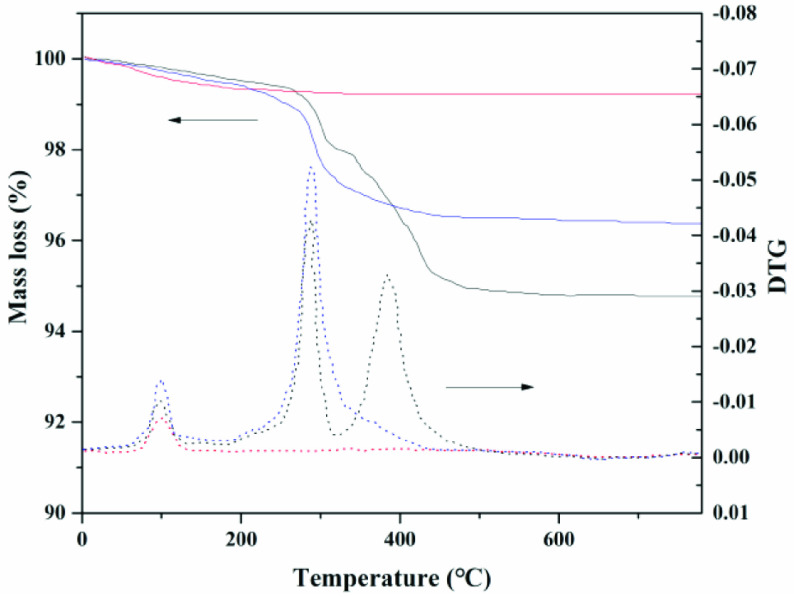
Thermogravimetric analysis/differential thermal gravimetry of immobilized catalyst.

The FT-IR spectra of the SG-NH2 (black line), choline derivative (blue line), and immobilized choline derivative (red line) were investigated and are shown in Figure 4. Two main bands were observed for the choline derivative: (i) the peak at 910–920 cm ^-1^ , attributed to the epoxy group, and a band peaking at 1000–1030 cm ^-1^ ascribed to the C-N of -N(CH _3_ ) _3_ [23]. After surface coupling, the new peak of SG-NH2 appeared at 2939 cm ^-1^ , attributed to the amino group, and the band at 2847 cm −1 was from the C-H stretching vibration [31]. After immobilization, the new peak of the immobilized choline derivative appeared at 3460 cm ^-1^ , attributed to the O-H due to the interaction between the amino group and epoxy group. Meanwhile, a peak near 1380 cm ^-1^ was a stretching vibration band of C-N-C, and a peak appearing near 947 cm ^-1^ was a characteristic peak of an alkyltrimethyl ammonium salt [32]. These results showed that the choline derivative was successfully immobilized on the surface of γ -aminated silica. Thermogravimetric analysis/differential thermal gravimetry (TG/DTG) results for silica (red line), SG-NH2 (blue line), and immobilized choline derivative (black line) are shown in Figure 5. In all cases, the weight loss from 80 °C to 120 °C corresponded to the removal of physically absorbed water. This was confirmed by the peak of DTG, in which the maximum mass loss rate of water was observed near 100 °C [33]. Compared with the purchased silica, the SG-NH2 had not only the weight loss peak of water but also the weight loss peak of the amino at 200–420 °C. The DTG analysis also indicated that the maximum mass loss rate of amino was at ~300 °C. A similar phenomenon can be found in previous studies [34,35]. On the other hand, the weight loss of the immobilized choline derivative was clearly divided into three stages by TG/DTG analysis: Region 1, the weight loss of absorbed water from 80 °C to 120 °C; Region 2, the weight loss of the amino group from 200 °C to 300 °C; Region 3: the weight loss of the choline derivative from 300 °C to 500 °C. Interestingly, the thermal stability of the choline derivative was stronger than that of amino group. The amino group was first broken, but the released choline derivative was not disrupted and volatilized, and it dropped in the sample cell. After the temperature reached 300 °C, the choline derivative was also cleaved. After immobilization, the stability of the primary ammonia was greater than that of the secondary ammonia. The rate of weight loss had an obvious decreasing trend, but the decline of about 10% was acceptable. Results indicated that the choline derivative was successfully immobilized on the surface of the γ -aminated silica.

### 3.3. Natural phospholipid transesterification reaction

**Figure 6:**
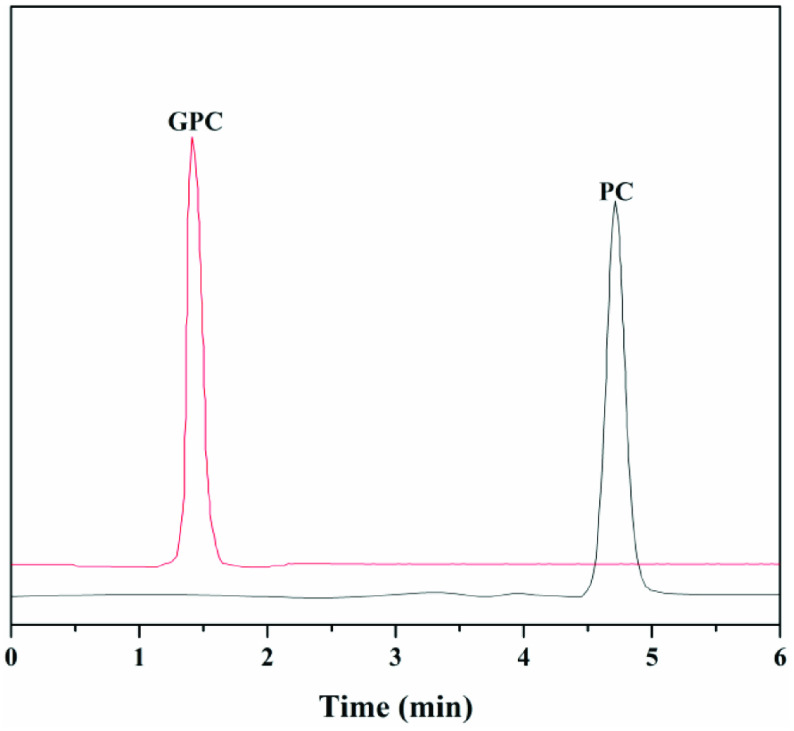
HPLC analysis of GPC from PC.

GPC was produced by hydrolysis of PC in transesterification reaction. After a 4-h reaction, the sharp peak at 4.7 min belonging to substrate PC completely disappeared and a new peak was detected at 1.4 min in the HPLC analysis, as shown in Figure 6. The retention time of the new product was the same as that of the standard sample of GPC, which was prepared as described [20].

#### 3.3.1. Effect of temperature

**Figure 7:**
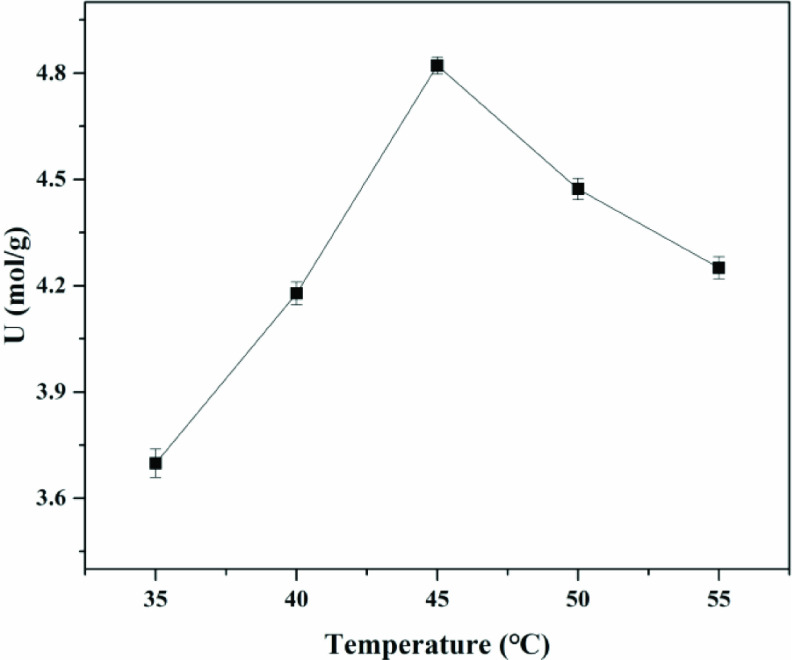
Effect of reaction temperature on the conversion. Catalyst amount - 20 g/L, time - 4 h, stirring speed - 250 1/min.

For transesterification procedures, temperature not only affected the reaction rate, but also affected the reaction thermodynamics and chemical kinetics. The transesterification activity of the immobilized choline derivative was measured at various temperatures, ranging from 35 to 55 °C, as shown in Figure 7. The optimum operational temperature of the immobilized choline derivative was 45 °C. The collisions between the immobilized choline derivative and the substrate increased with increasing temperature, so the energy of the substrate and the immobilized choline derivative also increased, which was beneficial for preparing GPC. However, the very high operational temperature aggravated the volatilization of the reaction solvents. In this work, ethanol was employed as the solvent. When the temperature exceeded 45 °C, an obvious declining trend in the reaction rate was observed due to the volatilization of the reaction solvents. In addition, the very high operational temperature required more energy consumption, increasing the production costs [14,16,36].

#### 3.3.2. Effect of stirring speed

**Figure 8:**
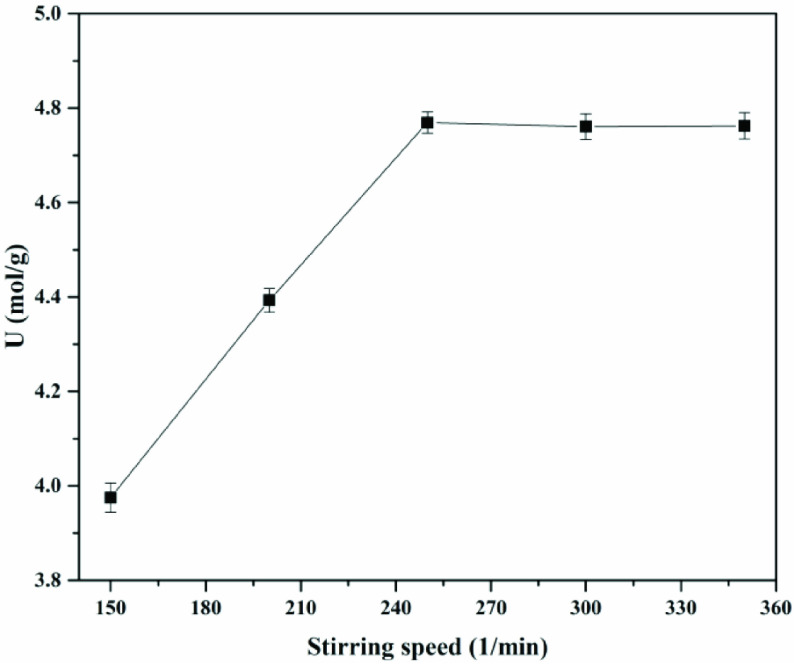
Effect of stirring speed on specific activity. Catalyst amount - 20 g/L, time - 4 h, temperature - 45 °C.

After immobilization, the mass transfer resistance must be taken into consideration. For the immobilized choline derivative, the stirring speed could reduce internal and external mass transfer resistances. The thickness of the boundary layer was decreased by the stirring speed increase to promote substrates and products to diffuse into and out of pore channels. Therefore, the stirring speed of the transesterification was systematically investigated and results are shown in Figure 8. It was observed that for stirring speeds below 250 1/min, the specific activity of the immobilized choline derivative increased with the stirring speed, while for stirring speeds above this value, no obvious increasing trend in specific activity was observed. Results indicated that the optimal stirring rate was 250 1/min [17,36].

#### 3.3.3. Effect of the amount of catalyst

**Figure 9:**
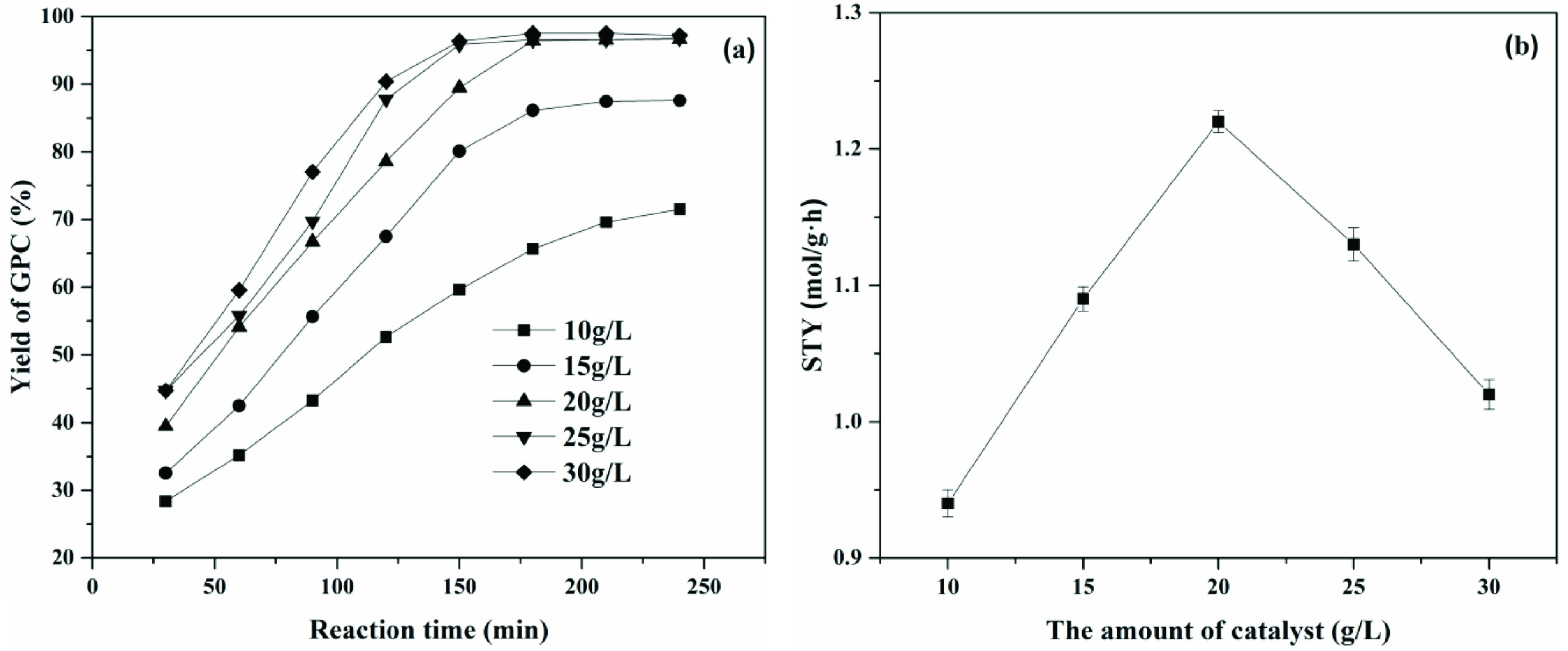
Effect of a) the amount of catalyst on specific activity and b) space-time yield. Time - 4 h, stirring speed - 250 1/min, temperature - 45 °C.

In order to obtain the highest production efficiency, the amount of catalyst was systematically investigated. As shown in Figure 9a, it was observed that for amounts of catalyst below 20 g/L, the conversion of PC increased with the amount of catalyst. When the amount of the catalyst exceeded 20 g/L, both the yield of GPC and the reaction rate barely changed. Next, the space-time yield [37] (STY, molGPC /gcatalyst h) under different amounts of immobilized choline derivative was investigated and results are shown in Figure 9b. When the amount of catalyst was 20 g/L, space-time yield was maximum and the yield of GPC also reached 97%. Therefore, the optimal amount of catalyst should be controlled at 20 g/L.

#### 3.3.4. Kinetic properties of free and immobilized choline derivative

**Figure 10:**
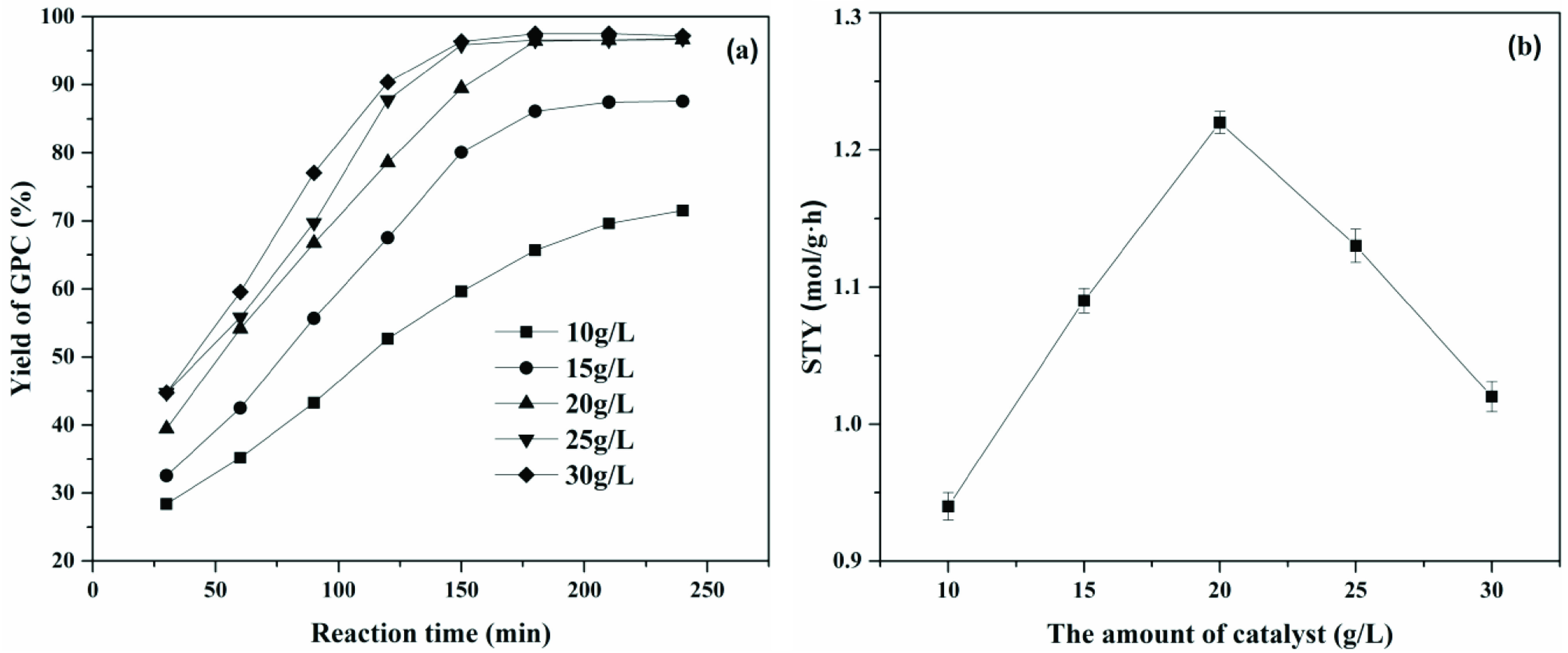
The transesterification formula of PC and ethanol.

Generally, the amount of ethanol in great excess relative to PC to maximize the yield, due to the low price of ethanol. The reaction process of transesterification is shown in Figure 10. The first-order kinetic model of Eq. (1) and the Arrhenius equation of Eq. (2) were employed to assess the reaction kinetics of transesterification and the relevant parameters (K and Ea) were calculated to help us thoroughly understand the reaction processes. As shown in the Table, an increase in reaction rate constants was seen in each reaction as temperature increased. The majority of the Pearson correlation coefficients were above 0.983, demonstrating a close approximation to the experimental data [38].

**Table:** The relevant parameters for transesterification reactions

Temperature, °C	Free choline		Immobilized choline derivative	
	Rate constant, k (min ^-1^ )	Pearson correlation coeff.	Rate constant, k (min ^-1^ )	Pearson correlation coeff.
20	0.0551	0.983	0.00439	0.985
25	0.0578	0.992	0.0049	0.986
30	0.061	0.995	0.00535	0.988
35	0.0655	0.99	0.00603	0.99
40	0.0694	0.984	0.00657	0.988
45	0.0732	0.986	0.00706	0.992
50	0.0752	0.985	0.00463	0.991

**Figure 11:**
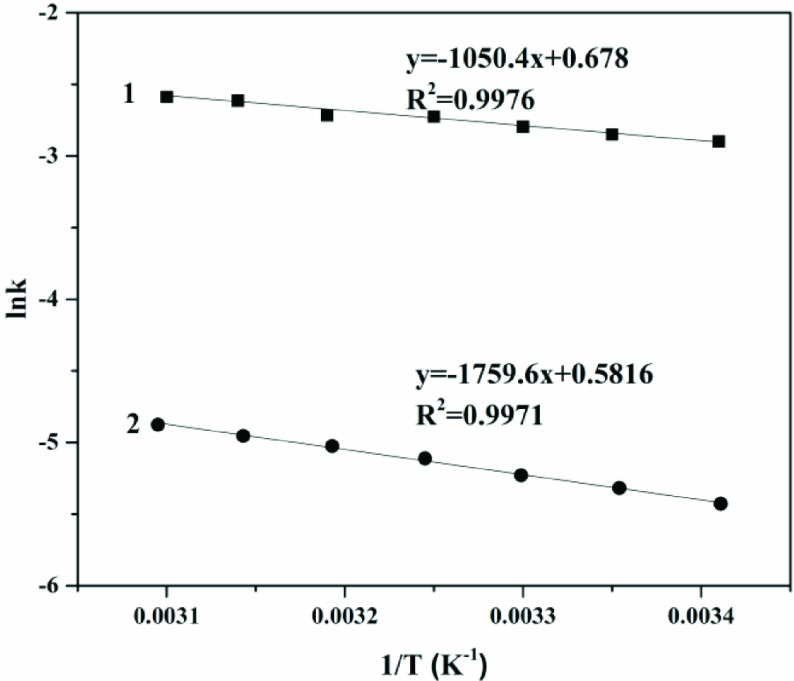
The plot of Arrhenius for link vs. 1/T, 1 - free choline, 2 - immobilized choline derivative.

The activation energy of the reaction was calculated via the Arrhenius equation (Eq. (2)). As shown in Figure 11, their values of R2 exceeded 0.997. The calculated activation energy of the immobilized choline derivative was 14.63 kJ/mol, which was slightly higher than that of free choline (9.73 kJ/mol). A reasonable explanation for this phenomenon is that the immobilization would bring mass transfer resistance for catalysts, which could be reduced by tailoring the reaction stirring speed, but not be eliminated completely. In this work, the increase was about 50% from free to immobilized choline derivative, which was accepted in the field of the immobilization [39,40].

#### 3.3.5. Comparison of free and immobilized choline derivative

**Figure 12:**
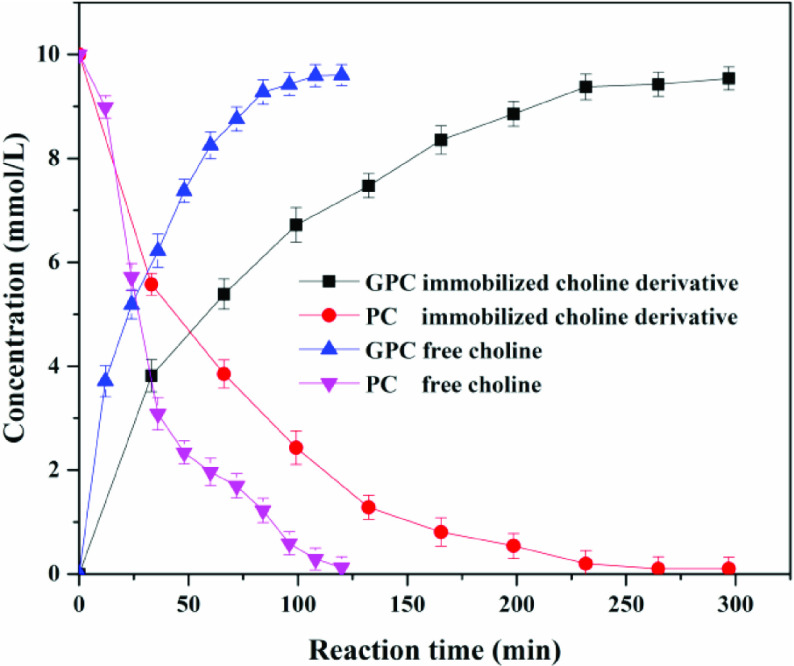
Reusability studies of catalyst, activating catalyst (blue), nonactivating catalyst (gray).

The reaction equilibrium times of the free and the immobilized choline derivative were investigated and results are shown in Figure 12. It was observed that the reaction equilibrium time of the free and immobilized choline derivative was 2 h and 4 h, respectively. The specific activity of free choline was 15.089 mol/g, which was approximately 3.1 times higher than that of the immobilized choline derivative (4.861 mol/g). However, high yields of GPC were obtained in all cases. For free and immobilized choline derivative, the yield of GPC reached about 97%.

**Figure 13:**
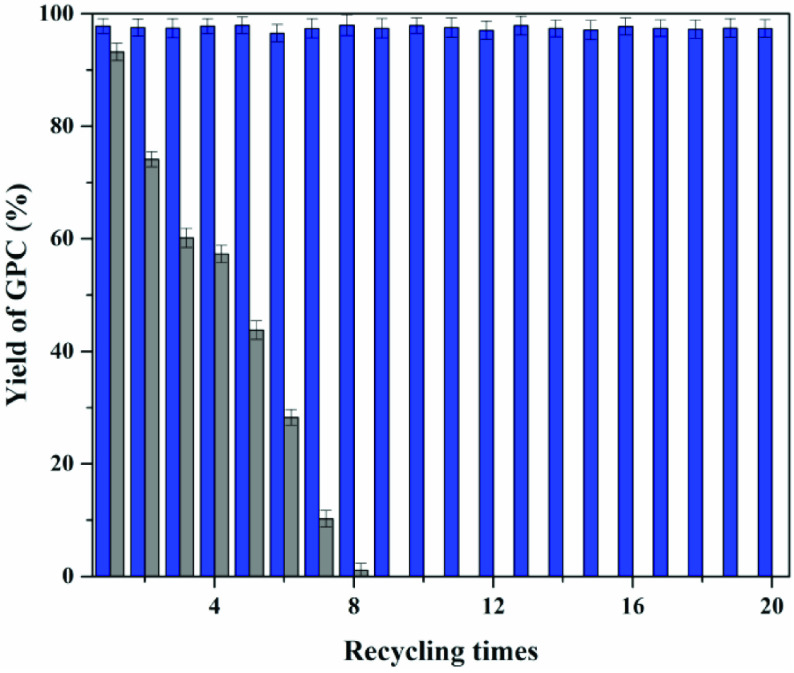
Effect of reaction time of free and immobilized choline derivative on the yield.

Next, the operational stability of the immobilized choline derivative was examined, as shown Figure 13. If the immobilized choline derivative was directly reused, 2.4% of the yield of GPC was obtained in the eighth batch. That might be caused by the combination between hydroxyl groups of the immobilized choline derivative and choline groups of GPC. Therefore, the catalyst should be treated by sodium hydroxide solution (0.01 M NaOH) after each batch reaction. The operational stability of these immobilized choline derivatives was further assessed. Results indicated that the recyclability of the immobilized choline derivative was excellent, as demonstrated in the 20 reaction cycles performed during the recycling experiment, which did not result in any loss of productivity. The calculated space-time yield of the immobilized choline derivative in recycling experiments reached 22.36 mol/g h, which was approximately 3 times higher than the free choline (7.55 mol/g h). After immobilization, the reaction rate was slightly decreased due to the mass transfer resistance, but the immobilized choline derivative could be easily collected and reused to increase the space-time yield and reduce the production cost significantly. Moreover, the immobilization also avoided choline derivative contamination of the product and improved the product safety considering the product GPC in food or medicine for human use.

## Conclusions

Choline derivatives were covalently immobilized on the surface of γ -aminated silica to prepare an immobilized choline derivative for the production of GPC. The catalyst contamination of the product can be completely avoided and, thus, the safety of the product will be increased significantly. The maximum yield of GPC reached 97.9%. The recyclability and stability of the immobilized choline derivative were excellent, as it was demonstrated that may be used 20 times without any loss of the productivity. The activation energy of the first-order kinetics was 14.63 kJ/mol. The excellent results make this immobilized choline derivative a more promising candidate for the industrial production of GPC.
